# Vitamin K in Vertebrates’ Reproduction: Further Puzzling Pieces of Evidence from Teleost Fish Species

**DOI:** 10.3390/biom10091303

**Published:** 2020-09-09

**Authors:** Silvia Beato, Francisco Javier Toledo-Solís, Ignacio Fernández

**Affiliations:** 1 Campus de Vegazana, s/n, Universidad de León (ULE), 24071 León, Spain; silviabeato@hotmail.it; 2 Consejo Nacional de Ciencia y Tecnología (CONACYT, México), Av. Insurgentes Sur 1582, Col. Crédito Constructor, Alcaldía Benito Juárez, C.P. 03940 Ciudad de Mexico, Mexico; fj.toledos@gmail.com; 3 Department of Biology and Geology, University of Almería, 04120 Almería, Spain; 4 Center for Aquaculture Research, Agrarian Technological Institute of Castile and Leon, Ctra. Arévalo, s/n, 40196 Zamarramala, Segovia, Spain

**Keywords:** K vitamers, steroid X receptor, vitamin K epoxide reductase, hypothalamus-pituitary-gonad axis, exosomes, non-coding RNAs, microRNAs, piRNAs

## Abstract

Vitamin K (VK) is a fat-soluble vitamin that vertebrates have to acquire from the diet, since they are not able to *de novo* synthesize it. VK has been historically known to be required for the control of blood coagulation, and more recently, bone development and homeostasis. Our understanding of the VK metabolism and the VK-related molecular pathways has been also increased, and the two main VK-related pathways—the pregnane X receptor (PXR) transactivation and the co-factor role on the γ-glutamyl carboxylation of the VK dependent proteins—have been thoroughly investigated during the last decades. Although several studies evidenced how VK may have a broader VK biological function than previously thought, including the reproduction, little is known about the specific molecular pathways. In vertebrates, sex differentiation and gametogenesis are tightly regulated processes through a highly complex molecular, cellular and tissue crosstalk. Here, VK metabolism and related pathways, as well as how gametogenesis might be impacted by VK nutritional status, will be reviewed. Critical knowledge gaps and future perspectives on how the different VK-related pathways come into play on vertebrate’s reproduction will be identified and proposed. The present review will pave the research progress to warrant a successful reproductive status through VK nutritional interventions as well as towards the establishment of reliable biomarkers for determining proper nutritional VK status in vertebrates.

## 1. Vitamin K Metabolites, Sources and Metabolism

Fat-soluble vitamins, including vitamin A, D, E, and K, are essential micronutrients for vertebrate’s development. In general, fat-soluble vitamins have a relatively heterogenic chemical structure, with most of them being terpenes or containing long terpene chains attached to an aromatic moiety. The exception is vitamin D (VD), which has a triene moiety instead of the aromatic one. Additionally, specific amounts and chemical forms of fat-soluble vitamins need to be provided in the diet, since these organic compounds cannot be endogenously synthesized by vertebrates, at least not in adequate amounts. Again, VD could be considered the exception. Although mammals are able to photoconvert 7-dehydrocholesterol into pre-vitamin D3 in the skin and only a minor supply is provided by the diet, no strong evidences of VD photosynthesis have been documented in fish species until now [1]. Furthermore, vitamin K (VK) can also be obtained from the intestinal microbiome, although the relevance of this contribution remains controversial [2]. Furthermore, no or low dietary toxicity was reported for VK in contrast to the other fat-soluble vitamins [1].

**Figure 1:**
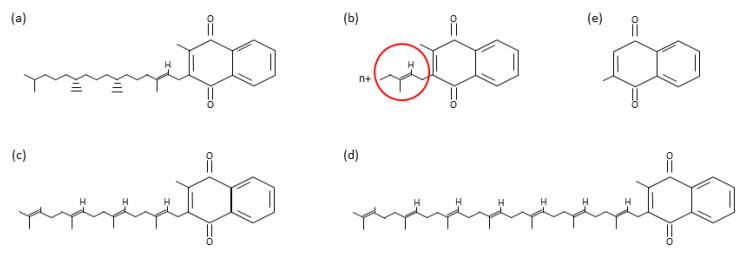
Main vitamin K metabolites. Phylloquinone (**a**), menaquinones, with the prenyl group highlighted with a red circle (**b**), menaquinone 4 (**c**), menaquinone 7 (**d**), and menadione (**e**).

In general, three different VK metabolites (or K vitamers) can be found (Figure 1). All of them are derived from quinone, exhibiting a common 2-methyl-1,4-naphthoquinone ring, but differing in the side chain at the C3-position [3]. Phylloquinone (or VK1), produced by photosynthetic plants, and menaquinones (or VK2), with microbial or animal origin, are the known VK natural compounds [4,5]. Regarding menaquinones, more than 20 different metabolites have been described, named MK-n accordingly to the number (n) of prenyl groups in the unsaturated side chain, but MK-4 and MK-7 being the most relevant forms from a nutritional point of view [5]. Menadione (or VK3) is the third K vitamer, a synthetic water-soluble salt without side chain. Despite being known to induce oxidative stress [6], it is categorized as safe VK supplement in livestock [7]. In fact, VK3 is easily excreted and shows lower bioavailability than the naturally occurring K vitamers. Nevertheless, although it is the most commonly used source of VK included in animal feeds, VK3 needs to be metabolized in order to be functional.

All of the K vitamers from the diet can be absorbed at the intestine, including those that originated from the intestinal microbiome [2,8]. Recent studies unequivocally evidenced the involvement of scavenger receptor class B type I (SR-BI), cluster of differentiation 36 (CD36), and Niemann-Pick C1-like 1 (NPC1L1) transporters on the uptake of VK1 at the intestinal lumen (reviewed in [9]). VK is absorbed chemically unchanged from the lumen at the proximal intestine, with 80% absorption efficiency for VK1 on its free form. Indeed, VK1 is the major circulating form of VK, although other K vitamers have also been found in the blood plasma (e.g., VK3, MK-4, and MK-7; [10,11]). The presence of VK3 is presumably resulting from a side chain cleavage of VK1 that only seemed to occur during intestinal absorption [11,12]. Unbound VK3 in circulation is highly reactive and easily excreted, not remaining in blood for a long period. The subsequent prenylation of VK3 to MK-4 at the enterocytes [11] or any other tissues (including testis; [13]) has been suggested, a reaction that seems to be catalyzed by the ubiquitously expressed UbiA prenyltransferase domain-containing protein 1 (Ubiad1) [8,14]. Nevertheless, orally administered MK-4 and MK-7 can be also converted to MK-4 [8] and thus, the previously described metabolic transformations of a certain amount of VK1 might be also applied to other members of the VK family [9].

As long as the VK was only considered to be involved on the hepatic γ-carboxylation of clotting factors, the liver was considered to be the uniquely significant storage site of VK [15]. Nowadays, it is known that mammalian liver stores 90 and 10% of menaquinones and phylloquinone [9], respectively. Other tissues/systems containing significant levels of K vitamers are blood plasma, adipose tissue, bone, heart, kidney, spleen, brain, ovaries and/or testes, being thyroid, adrenal, testes, ovaries, pancreas, salivary, and skin particularly enriched in MK-4 [9,16]. Such wide tissue distribution is consistent with the ubiquitous and evolutionary conserved expression of the main drivers of VK pathways (briefly described in the next subsection): the *pregnane X receptor* (*pxr*; also known as *steroid X receptor* or *sxr*) [17,18,19,20] and the *γ-glutamyl carboxylase* (*ggcx*) [21,22]. Furthermore, MK-4 enriched distribution in particular tissues seemed to be determined by local synthesis of MK-4, rather than by its uptake [23]. In this sense, while VK1 is known to be preferentially retained in the liver to assist γ-carboxylation of clotting factors, where VK recycling is basically performed through VK epoxide reductase (VKOR) complex subunit 1 (VKORC1); VK2 have to be redistributed through circulation (or in situ converted) in order to be available for extra-hepatic tissues, where VK recycling could be more related to VKORC1-like protein 1 (VKORC1L1) [24]. Indeed, several studies suggested VK2 as being more effective in activating extra-hepatic VKDPs than VK1 [25]. Nevertheless, levels of each K vitamers at each tissue/system might depend on the considered vertebrate species, gender, population, and/or the daily dietary uptake [1,16,26]. Furthermore, a variable response to VK1 dietary supplementation in humans was recently associated to differences in DNA methylation in multiple genomic regions [27].

Because VK stores are very labile and rapidly respond through specific pathways to nutritional status (fasting/feeding/nutritional supplementation), as well as other conditions, such as pharmacological treatment with VK anticoagulants [2,16,20,28,29,30,31], an optimized daily VK dietary uptake is recommended [32,33]. This daily uptake may avoid VK deficiencies and their related pathological conditions. To fulfill VK nutritional requirements, an equilibrated intake of VK from diverse dietary sources and a healthy intestinal microbiota seems to be necessary [2]. Determining a general optimal dietary VK intake seemed to be pursuing a chimera, since a reliable golden standard biomarker of VK nutritional status is still lacking [33,34]. In this sense, identifying the biological functions that require VK is critical. Nowadays, VK is quite well known in vertebrates to be required for blood coagulation [35,36,37], skeletal and joint tissues [19,38,39,40,41,42], and pathological calcification and inflammation [37,43,44,45]. There are some reports on their potential role on redox homeostasis [41,46,47], sphingolipid [48,49] and glucose metabolism [50], neural development and cognitive capacities [20,48,51,52], as well as angiogenesis [53]. Furthermore, some evidences have been presented regarding the potential role of VK on the reproduction of mammalian [54,55] and fish species [37,49,55]. Altogether, the idea of a well conserved function of VK along evolution is reinforced, including the related molecular pathways [56,57].

## 2. Vitamin K Molecular Pathways

**Figure 2:**
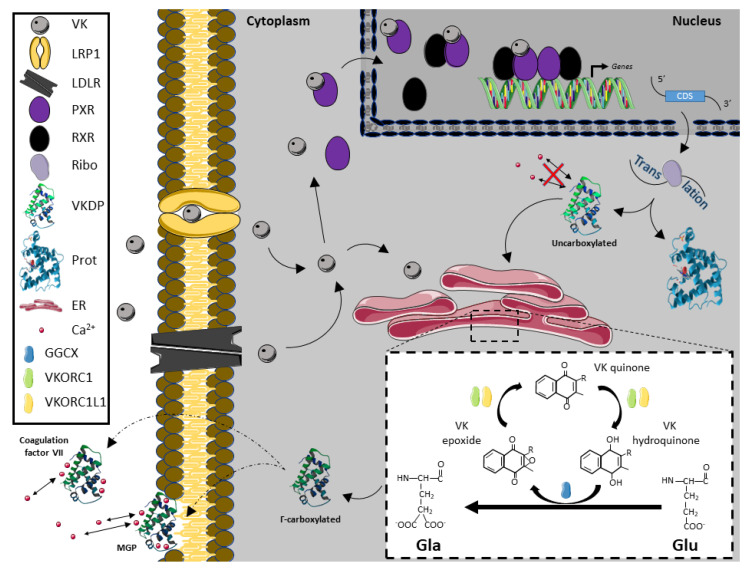
Vitamin K metabolic and transcriptional roles. Vitamin K (VK) enters cell cytoplasm through the action of low-density lipoprotein receptor (LDLR) and prolow-density lipoprotein receptor-related protein 1 (LRP1). VK might transactivate pregnane X receptor (PXR), which in turn form an heterotetramer with the retinoid X receptor (RXR) able to be bound to the DNA RXR-PXR response element sequences and recruit co-activators and RNA polymerase II in order to transcribe the target genes. In this manner, PXR is able to activate the transcription of different genes, including known VK dependent proteins (VKDPs), as such MGP, among others. After genes encoding VKDPs are transcribed, mRNAs are translated to the corresponding proteins. VKDPs can undergo γ-carboxylation at the endoplasmic reticulum (ER), where VK (quinone form) is reduced to VK hydroquinone which acts as a co-factor of the γ-glutamyl carboxylase (GGCX) enzyme to γ-carboxylate those VKDPs. As a result of the conversion of Glu residues in Gla residues, VKDPs are able to bind calcium ions (Ca^2+^) while VK hydroquinone is oxidated to VK epoxide. Both VK epoxide reductase complex subunint 1 (VKORC1) and VKORC1-like protein 1 (VKORC1L1) enzymes are able to reduce VK epoxide to VK quinone and VK quinone to VK hydroquinone; therefore recycling VK. *CDS*, coding sequence; *ER*, endoplasmic reticulum; *GGCX*, γ-glutamyl carboxylase; *Gla*, γ-carboxylated glutamic acid; *Glu*, glutamic acid; *LDLR*, low-density lipoprotein receptor; *LRP1*, low-density lipoprotein receptor-related protein 1; *MGP*, matrix Gla protein; *Prot*, protein; *PXR*, pregnane X receptor; *Ribo*, ribosomal subunits; *RXR*, retinoid X receptor; *VK*, vitamin K; *VKDP*, VK dependent protein; *VKORC1*, VK epoxide reductase complex subunit 1; *VKORC1L1*, VKORC1-like protein 1.

In addition to its potential antioxidant function, VK may exert their biological functions by two different molecular pathways: acting as a co-factor of GGCX and transactivating the NR PXR. How VK might be up taken, metabolized, transported, and exert their effects at a transcriptional level or through the carboxylation of VKDPs in vertebrates is summarized in Figure 2, based on the most recent literature [1,9,15,17,29,58,59,60,61,62].

GGCX is a transmembrane protein that is localized at the endoplasmic reticulum (ER) and it catalyzes the conversion of specific glutamate (Glu) residues to γ-carboxyglutamate (Gla) residues, a process called γ-carboxylation, where VK acts as a co-factor [62]. GGCX activity was detected in most or all mammalian tissues, and the carboxylase gene has been demonstrated to be evolutionarily conserved in other vertebrates, such as fish, but also in chordates (such as *Ciona intestinalis*) and invertebrates [9,22,63,64,65,66]. Γ-carboxylation is vital for the survival of vertebrates as mouse lacking GGCX die at birth due to fatal hemorrhages [35]. Indeed, as a co-factor of the GGCX enzyme, VK is essential for the activation and proper functioning of multiple proteins that are known as VK-dependent proteins (VKDPs). The most well-known VKDPs are those that are involved in blood clotting: coagulation factors (FII, FVII, FIX and FX) and natural anti-clotting agents (protein C, protein S and protein Z) [36]. However, GGCX also catalyzes γ-carboxylation of other VKDPs involved in various biological processes, such as inflammation (gla-rich protein (GRP)), bone formation (osteocalcin (OC; also known as bone Gla protein (BGP or BGLAP))), cell proliferation (growth arrest-specific 6 (GAS6)), and soft tissue mineralization (matrix Gla protein (MGP)) [36,67]. Of note, while VKDPs involved in blood coagulation are γ-carboxylated at the liver, the other VKDPs are γ-carboxylated in extra-hepatic tissues. An abnormal rate of γ-carboxylation at extra-hepatic tissues might have relevant physiological implications, from altered blood coagulation and skeletal development to impaired gametogenesis.

Carboxylation requires the abstraction of a proton from the four-carbon of the glutamate residue by reduced VK, which results in the conversion of VK into VK epoxide. The VK epoxide must be recycled, a reaction that is catalyzed by two VK 2,3-epoxide reductases (VKORs) [62]. VKORs are also transmembrane proteins that are localized at the ER, being essential for a continuous γ-carboxylation of VKDPs, and intimately connected to GGCX. Indeed, although VKORC1 knockout mice survive longer than GGCX^-/-^ mice, they exhibited early postnatal lethality due to severe bleeding [68], which suggests VK recycling is critical in sustaining γ-carboxylation of blood clotting factors. A well evolutionary conserved structure and function of VKORs has been suggested [57], where the VKORC1 and VKORC1L1 paralogs probably arose from the duplication of a VKOR ancestor gene [29,30,31,46,56,57,60,61]. A ubiquitous gene expression of both *vkorc1* and *vkorc1l1* genes in vertebrates has been reported [29,30,31,56,60,61]. In vertebrates, the highest *vkorc1* expression is found in liver, consistent with being the main isoform that is responsible for recycling VK epoxide originated from hepatic γ-carboxylation of hemostasis-related VKDPs [60,61]. In contrast, highest *vkorc1l1* gene expression in vertebrates was found in brain and ovaries, but also high expression levels were reported in organs, like kidney, eye, and testis [31,56,58]. Recent structural insights on how VKORC1 is preferentially binding to VK1 and MK4, rather than MK7 [69] pointed out further differences on VK recycling capacity, depending on the substrate (VK form) and the VKOR paralog (VKORC1 or VKORC1L1). The presence of VK metabolites, γ-carboxylation activity, and VK recycling in male and female gonads might be the first evidence suggesting that VK might be required in vertebrate’s reproduction.

The other main pathway of the VK is the transactivation of the NR PXR [17]. PXR is a member of the NR super-family NR1I (NR1I2), including PXR, vitamin D receptor (VDR, NR1I1), and constitutive androstane receptor (CAR, NR1I3) [70]. Molecular phylogenies of the NR1I super-family suggested that VDR, PXR, and CAR sequentially evolved from a common ancestor represented by *Ciona intestinalis* VDR/PXR [20]. As a NR, PXR modulates the transcription of genes showing specific response elements (REs) on their promoter regions to which it binds through their DNA binding domain (DBD), once it is activated by known ligands [71]. In contrast to the DBD, its ligand binding domain (LBD) exhibited an evolutionary low degree of conservation [71] and thus, PXR ligands might differ across species [71,72]. PXR signaling cascade regulations seem to be further puzzling, as PXR subcellular localization and transcriptional activity are also dependent on multiple post-translational modifications, including phosphorylation, acetylation, SUMOylation, and ubiquitination [73]. Our understanding on how PXR function in vertebrate species other than mammalian species is still limited. Several studies have suggested as VK and warfarin might bind to mammalian’s PXR as ligands, using a diverse set of methodological approaches [17,71,74]. However, only in vivo and in vitro transcriptional approaches suggest, as fish PXR transcriptional activation through these compounds might be evolutionary retained from teleosts [20,37].

In mammals, PXR is mainly expressed in liver and intestine, where the PXR-mediated gene activation program is primarily recognized to increase drug metabolism, drug transport, and drug efflux pathways in these tissues. However, the control of retinoid metabolism, inflammation, iron, glucose, lipid, cholesterol, bile acid, and bilirubin homeostasis, as well as the regulation of bone mineral density, were also demonstrated [19,70,75,76]. In this sense, mammalian *pxr* transcripts have also been detected in osteoblastic and neural cells, as well as in the endothelial cells of the blood-brain barrier (BBB) [17,18,19,77]. In fish, a broad distribution of *pxr* expression in tissues/organs was reported in different species [20,37,78,79]. Fish *pxr* transcripts were specifically detected at brain (granular layer), eyes (inner nuclear and nerve fiber layers as well as rod nucleus), cartilage (cell rich hyaline cartilage), bone, intestine, liver, gonads, spleen, and kidney [20,80], widening the potential implication of PXR signaling in the development and physiological status of vertebrates, including its reproductive performance.

## 3. The Impact of Vitamin K in Reproduction

Reproduction is a tightly regulated biological process that guarantees species perpetuation and increases intra-specific genetic variability, allowing for offspring to increase its fitness and adaptation to the environmental changes and avoiding the extinction. Although vertebrates exhibit a diverse set of modes of sexual reproduction (including hermaphroditism), with oviparous, ovoviviparous, or viviparous offspring production, semelparity or iteroparity (with single (seasonal), or multiple reproductive cycles along the year), as well as with synchronous, group-synchronous, and asynchronous gonadal development; it requires a myriad of precisely orchestrated events, governed by host signaling pathways that are considerably conserved along its evolution [81]. A great research effort has been paid to understand the physiological changes and the related underpinning molecular pathways in order to warrant successful reproduction and solve reproductive disorders. Consequently, it is known that reproductive fitness may be influenced by life conditions, as obesity, stress, environmental pollution, and/or daily dietary intake of particular nutrients [82,83,84,85]. Although reproductive system development and regulation is a continuous process, in the present article we will review (i) the gonad development, (ii) the hypothalamus-pituitary-gonad (HPG) axis regulation, and (iii) the tissue crosstalk with relevant organs and signaling pathways separately, in order to simplify the highly complex picture. Along the description of such processes, the different pieces of evidence showing the puzzling impact of VK on vertebrate’s reproduction will be highlighted, supporting the role of VK in this process, in addition to the known association between protein Z (a VKDP with extrahepatic γ-carboxylation) and pregnancy outcome in humans (reviewed in [86]).

### 3.1. Direct Impact of Vitamin K on the Gonads

**Figure 3:**
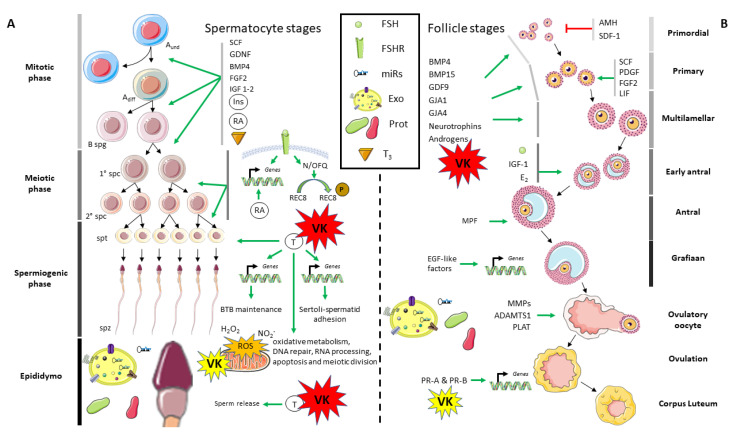
Gonad development and related molecular pathways in vertebrates. General overview of spermatogenesis (**A**) and oogenesis (**B**), specifying some of the most relevant pathways. The particular mechanisms by which vitamin K (VK) has a demonstrated effect on gonad development are highlighted in red, while those showing potential evidences are highlighted in yellow. *ADAMTS1*, a disintegrin-like and metallopeptidase with thrombospondin type 1 motif, 1; *A_diff_*, differentiated spermatogonia A; *AMH*, anti-Müllerian hormone; *A_und_*, undifferentiated spermatogonia A; *B spg*, spermatogonia B; *BMP4*, bone morphogenetic protein 4; *BMP15*, bone morphogenetic protein 15; *BTB*, blood-testis barrier; *E_2_*, estradiol; *EGF-like factors*, epidermal growth factor-like factors; *FGF2*, fibroblast growth factor 2; *FSH*, follicle-stimulating hormone; *FSHR*, follicle-stimulating hormone receptor; *GDF9*, growth differentiation factor 9; *GDNF*, glial cell-derived neurotrophic factor; *GJA1*, gap junction alpha-1 protein; *GJA4*, gap junction alpha-4 protein; *IGF 1-2*, insulin like growth factor 1 and 2; *Ins*, insulin; *LIF*, leukemia inhibitory factor; *MMP*, matrix metalloproteinases; *MPF*, maturation-promoting factor; *N/OFQ*, nociceptin/orphanin FQ; *P in brown circle*, phosphorylated protein; *PDGF*, platelet-derived growth factor; *PLAT*, tissue-type plasminogen activator; *PR-A*, progesterone receptor A; *PR-B*, progesterone receptor B; *RA*, retinoic acid; REC8, meiotic recombination protein REC8 homolog; *SDF-1/CXCL12*, stromal cell-derived factor-1; *SCF*, stem cell factor; *spt*, spermatid; *spz*, spermatozoa; *1° spc*, primary spermatocyte; *2° spc*, secondary spermatocyte; *T*, testosterone; *T_3_*, triiodothyronine.

Gonad development and maturation is a process that is controlled through different signaling pathways at the local level (Figure 3). In males, spermatogenesis can be generally divided in three different phases: mitotic, meiotic, and spermiogenic. During the mitotic phase, the undifferentiated spermatogonia A, through the action of a well-defined and diverse set of growth factors and molecules (including stem cell factor (SCF), glial cell-derived neurotrophic factor (GDNF), bone morphogenetic protein 4 (BMP4), fibroblast growth factor 2 (FGF2), insulin like growth factor 1 and 2 (IGF-1 and 2), insulin (Ins), retinoic acid (RA), and triiodothyronine (T_3_)), differentiates and leads to the formation of the spermatogonia B, which is characterized by a more rapid mitotic division potential [87,88,89,90]. At the meiotic phase, primary and secondary spermatocytes differentiate and proliferate under the control of follicle-stimulated hormone (FSH). In order to directly induce spermatocytes meiosis, in Sertoli cells, FSH binding to its receptor leads to the activation of the gene expression of *neuregulin 1* and *3* (*nrg1* and *3*, respectively). Similar to FSH, retinoic acid (RA) also has the capacity to induce the transcription of both *nrg1* and *3*, through the binding to its own receptors, as demonstrated in Nrg1 ^Ser−/−^mutant mice (reviewed in [89]). In addition, FSH up-regulates nociceptin (N/OFQ), which promotes the phosphorylation of the meiotic recombination protein REC8 homolog (REC8), triggering chromosome dynamics during spermatocyte meiosis (reviewed in [89]). Testosterone, through canonical and non-canonical signaling pathways, regulates different and key biological processes that are required for proper spermatid maturation, such as oxidative metabolism, DNA repair, RNA processing, apoptosis, and/or meiotic division, as demonstrated in murine models under testosterone suppression (reviewed in [91]). In particular, testosterone is needed in order to initiate the transcription of *occluding* and *claudins 3* and *11*, in order to warrant blood-testis barrier (BTB) maintenance, as well as the one of *cadherins*, *integrins*, and *laminins* to form protein complexes to attach Sertoli cells to elongated spermatids.

Nowadays, it is clear that both main molecular pathways of VK, PXR transcriptional activation, and γ-carboxylation, are present in main tissues involved in vertebrate’s reproduction, the brain and the gonads. Transcripts of *pxr*, *ggcx*, and *vkors* were specifically found in the pituitary, testis, and ovaries in different vertebrate species, from fish to mammals [18,20,22,31,37,56,78,80,92,93]. Although, in mammals, PXR can be transactivated by molecules other than VK and VK anticoagulants, like warfarin, as well as its specific ligands may differ from fish to mammalian species [71,94], transcriptional activation of *cyp3a1* through PXR by VK might be a key step on the control of progesterone, cholesterol, and testosterone synthesis [95] and thus, of the reproductive system, as demonstrated by in silico and in vitro assays using primary rat hepatocytes. *Pxr*-deficient rodents shows poor breeding success [96]. Furthermore, the activation of PXR in humans and rodents leads to the transcription of genes encoding phase I enzymes, including *cyp3a4, cyp3a23, cyp3a11, cyp2b6, cyp2b9, cyp2c55, cyp2c8, cyp2c9, cyp2c19* and *cyp1a*, but also phase II enzymes (eg. *UDP-glucuronosyl transferase* (*ugt*), *sulfotransferase* (*sult*), and *glutathione S-transferase* (*GST*) enzymes), both controlling the synthesis and metabolism of steroids such estrogen (E_2_), among other compounds (reviewed in [97]); being particularly important CYP3A4 and SULT2A1, known to act on the metabolic deactivation of androgens [98]. In this sense, the transactivation of PXR in porcine Leydig cells increased the expression of *cytochrome B5A* and *cytochrome B5 reductase 1*, as well as *hydroxysteroid (17-beta) dehydrogenase 4* and *retinol dehydrogenase 12* genes that are involved in steroidogenesis [99]. The undoubtedly demonstration of VK playing a key role on reproduction might come from nutritional studies. Rats fed with VK deficient diets had low expression of *cyp11a*—a rate-limiting enzyme in testosterone synthesis—where mRNA levels were positively correlated with the MK-4 concentration in testis [54]. The effect of VK on testosterone levels (at testis and/or plasma) seemed to be evolutionary conserved between fish and mammals, as increasing the dietary VK levels increased the testosterone levels in both mammalian [54,55] and fish species [49]. The impact of VK on spermatogenesis through the exposure to warfarin (a VK recycling inhibitor) was further described recently. Sprague–Dawley rats fed with a diet containing warfarin had a delayed spermiation, presence of multinucleated giant cells in the seminiferous tubules, germ cells degeneration, asthenozoospermia, oligozoospermia, increased percentage of abnormal sperm morphology, and lower sperm concentration and motility when compared to the controls [100]. These results on the rat testis were correlated not only with the lower serum testosterone level, but also with a higher serum LH level [100].

It seems like VK has still conserved its role as an important antioxidant molecule from invertebrates. Because spermatozoa have limited antioxidant defense mechanisms and a limited capacity for detection and repair of DNA damage, these cells are among the most vulnerable to oxidative stress. Indeed, oxidative stress has been linked to male infertility, inducing reduced sperm motility, sperm DNA damage, and increased risk of recurrent abortions and genetic diseases, although certain levels of reactive oxygen species (ROS) are required for the maturation of spermatozoa, acrosome reaction, capacitation, hyperactivation, and spermatocyte fusion in humans (reviewed in [101]). Although oxidative status was not specifically determined in Senegalese sole (*Solea senegalensis*) broodstock fed control and dietary VK1 supplemented diets during six months, Fernández and colleagues [49] found lower sperm DNA fragmentation in Senegalese sole males fed supplemented diet, in line with an improved antioxidant capacity of juvenile Jian carp (*Cyprinus carpio* var. Jian) fed increased levels of dietary VK [102], and the in vitro elimination of intracellular ROS through VKORC1L1 in the presence of VK [46]. As previously mentioned, *vkorc1l1* was found to be expressed in the testis of vertebrates, including rodents [60] and Senegalese sole [31]. Furthermore, VKORC1L1 KO cells are sensitive to oxidative stress, while VK1 supplementation seemed to compensate their cell stress [103]. Thus, VK1 and/or VK2 dietary supplementation might be another antioxidant therapy to prevent male infertility through ROS, as has been already demonstrated for vitamin C, vitamin E, selenium, zinc, and glutathione in humans (reviewed in [101]).

In mammals, spermatozoa will mature at epididymis through the interaction with different molecules, proteins, and microRNAs (miRs) (included in exosomes or not), and finally induced to be released by testosterone [91,104,105]. In addition to the regulation of testosterone synthesis by VK (above-mentioned), VK might also affect spermatogenesis through the post-transcriptional regulation of miRs (please, see the specific section of tissue crosstalk in this sense).

In females, the gametogenesis process is also quite conserved along evolution, and the detailed review performed by [106] in mammalian gametogenesis is highly recommended for a deeper lecture. In mammals, the first steps of folliculogenesis (from primordial to multilamellar follicles) are known to be gonadotropins-independent phases. Anti-Müllerian hormone (AMH) and stromal cell-derived factor-1 (SDF-1/CXCL12) inhibit primordial follicle recruitment (reviewed in [107]). In contrast, while stem cell factor (SCF), platelet-derived growth factor (PDGF), fibroblast growth factor 2 (FGF2), and leukemia inhibitory factor (LIF) specifically promote the transition from primordial to primary follicles [106], different growth factors, including bone morphogenetic protein 4 (BMP4) and 15 (BMP15), growth differentiation factor 9 (GDF9), gap junction alpha-1 protein (GJA1, also known as Connexin 43), gap junction alpha-4 protein (GJA4, also known as Connexin 37), neurotrophins and/or androgens, stimulate oocyte growth, granulosa, and theca cells proliferation. The effect of VK on male gametogenesis might also occur in female gametogenesis, where a dietary VK supplementation also increased the plasma testosterone levels in Senegalese sole females [49]. If VK also regulates the synthesis of steroids in ovaries through PXR, VK nutritional status might also affect multilamellar and early antral follicles recruitment and ovulation. Furthermore, the detected expression of *vkorc1* and *vkorc1l1* as well as *ggcx* in male and female gonads suggest that VK is required and recycled for vertebrate’s reproduction [31,56,92]. Nevertheless, it still remains to uncover whether i) the local γ-carboxylation of VKDPs and/or VK antioxidant function is the required pathway for assuring reproductive success or ii) if the transactivation of PXR through VK is the main driver pathway of VK effects on gametogenesis.

During the mammalian early antral and antral follicle maturation with a continuous granulosa cells proliferation, insulin like growth factor 1 (IGF-1), and E_2_, in addition to FSH, play an important role. Only the most mature antral follicles (most sensitive to FSH action) go on towards its maturation until the dominant follicle recruitment. LH surge-induced factors are essential for the oocyte meiotic resumption, cumulus expansion, until the ovulation, and luteinization in mammals. In this sense, maturation-promoting factor (MPF) leads to oocyte meiotic resumption, while epidermal growth factor-like factors (EGF-like factors) activate the expression of *prostaglandin synthase 2* (*ptgs2*), *hyaluronan synthase 2* (*has2*), and *tumor necrosis factor* (TNF) *α-induced protein 6* (*tnfaip6*) in order to the cumulus expansion to take place. For follicle rupture in mammals, the action of matrix metalloproteinases (MMPs), tissue-type plasminogen activator (PLAT), and a disintegrin-like and metallopeptidase with thrombospondin type 1 motif (ADAMTS1) is required [106]. In the meanwhile, mammalian oocytes interact with different proteins, metabolites, and miRs packaged in exosomes that are present in the follicular fluid’s [105,108], being finally ovulated upon the transcriptional activation of *endothelin 2* (*edn2*), *cGMP-dependent protein kinase 2* (*prkg2*), *interleukin 6* (*il6*), and *synaptosome associated protein 25* (*snap25*) genes through the action of progesterone receptors (PR-A and PR-B; reviewed in [106]). Because PXR control the steroid synthesis and metabolism, including progesterone, this might represent another mechanism by which VK might influence progesterone receptors transactivation and thus, ovulation, which remains to be elucidated.

Oxidative stress may also affect ovaries, being ROS involved in the initiation of apoptosis in antral follicles caused, for instance by several chemical and physical agents in mammalian species (reviewed in [109]). Antral rat follicles suffered apoptotic death without gonadotropin support, while the capacity of FSH to suppress it was strongly associated to follicular glutathione (GSH) synthesis. In cultured rat ovarian follicles, the antioxidants superoxide dismutase (SOD), catalase, *N*-acetyl cysteine and ascorbic acid, are all able to inhibit apoptosis in antral follicles in the absence of FSH [110]. Thus, VK might also protect primordial and primary follicles from death if its antioxidant role in vertebrates is confirmed. In this sense, an increased oxidative stress response (at transcriptional level) in zebrafish (*Danio rerio*) embryos under VK-induced deficiency was found [41]. Nevertheless, the potential preventive antioxidant role of VK1 and VK2 is not observed for its analog, the menadione (VK3). It is known that while in vivo treatment of neonatal mice with increasing menadione doses decreased the total number of follicles per section, in vitro cultured ovaries exposed to menadione showed oxidative DNA lesions and activated caspases, probably through lipid peroxidation [6].

In addition to the potential effects of VK on testosterone synthesis and the antioxidant status, VK might also affect gametogenesis through other particular pathways, such as some of the VKDPs and its carboxylation status. In this sense, protein S (PROS1) and GAS6 are also expressed in the testis of Sprague–Dawley rats [100], and both PROS1 and GAS6 act as specific ligands for the TAM (TYRO, AXL and MERTK) family of receptor protein tyrosine kinases that play an important role on reproductive system (reviewed in [111]), among other tissues such as brain (see below). Because VK deficiency might impair the action of those VKDPs through its carboxylation status, the altered intracellular Ca^2+^ homeostasis in particular tissues (such as testis) due to the limited carboxylation of these VKDPs, might be an additional relevant pathway on reproduction.

### 3.2. The Vitamin K Impact on the Hypothalamus-Pituitary-Gonad Axis

**Figure 4:**
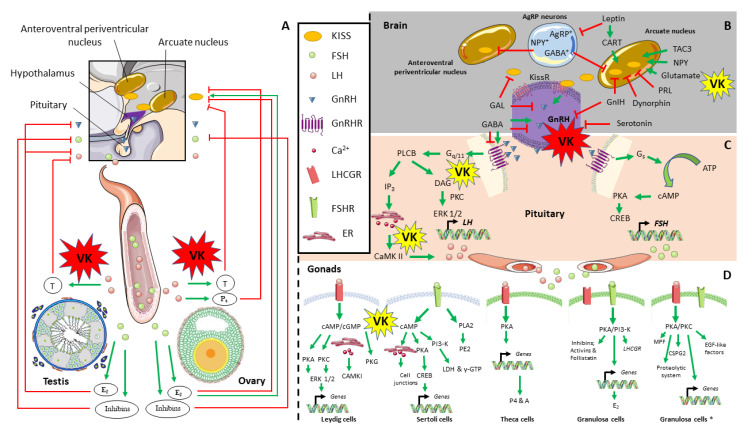
Hypothalamus-pituitary-gonad (HPG) axis in vertebrates and related molecular pathways. General hormonal control of the HPG axis, from neural kisspeptin stimulation to gonadal testosterone and androgens feedback regulation (**A**). Hormonal control of GnRH synthesis and release at brain (**B**). GnRH signaling pathway for FSH and LH synthesis (**C**). FHS and LH signaling pathway at specific cells from testis and ovaries: Leydig, Sertoli, Theca, Granulosa cells (**D**). The particular mechanisms by which vitamin K (VK) has a demonstrated effect on HPG axis are highlighted in red, while those showing potential evidences are highlighted in yellow. The *asterisk* indicates Granulosa at pre-ovulation stage, whereas *green arrows* and *red* lines indicate stimulatory and inhibitory activity, respectively. The symbol + indicates cells expressing particular genes. *A*, androstenedione; *AgRP*, agouti-related peptide; *ATP*, adenosine triphosphate; *CAMK I*, calcium/calmodulin kinase I; *CAMK II*, calcium/calmodulin-dependent kinase II; *cAMP*, cyclic adenosine monophosphate; *CART*, cocaine- and amphetamine-regulated transcript; *cGMP*, cyclic guanosine monophosphate; *CREB*, cAMP response element (CRE) binding protein; *CSPG2*, chondroitin sulfate proteoglycan 2; *DAG*, diacylglycerol; *E_2_*, estradiol; *EGF-like factors*, epidermal growth factor-like factors; *ERK 1/2*, mitogen-activated protein kinase; *FSH*, follicle-stimulating hormone; *FSHR*, follicle-stimulating hormone receptor; *GABA*, gamma-aminobutyric acid; *GAL*, galanin; *Γ-GTP*, γ-glutamyl transpeptidase; *GnIH*, gonadotropin inhibitory hormone; *GnRH*, gonadotropin releasing hormone; *GnRHR*, gonadotropin releasing hormone receptor; *GPRC6A*, G protein-coupled receptor class C group 6 member; *IP3*, inositol trisphosphate; *KISS*, kisspeptin; *KISSR*, kisspeptin receptor; *LDH*, lactate dehydrogenase; *LH*, luteinizing hormone; *LHCGR*, luteinizing hormone/choriogonadotropin receptor; *MPF*, maturation-promoting factor; *NPY*, neuropeptide Y; *P4*, progesterone; *PI3-K*, phosphatidylinositol 3-kinase; *PKA*, protein kinase A; *PKC*, protein kinase C; *PKG*, protein kinase G; *PLA2*, phospholipase A2; *PLCB*, phospholipase Cβ; *ER*, endoplasmic reticulum; *T*, testosterone; *TAC3*, tachykinin-3.

In vertebrates, the HPG axis control gametogenesis and reproduction performance in a tightly regulated manner (Figure 4). In females, at the top of the axis, anteroventral periventricular and arcuate nucleus-derived kisspeptin (KISS) induces the release of gonadotropin-releasing hormone (GnRH) at the hypothalamus [112], which, in turn, stimulates gonadotropic cells to synthetize and secrete luteinizing hormone (LH) and follicle-stimulating hormone (FSH) in the anterior pituitary [113]. Recent studies in zebrafish also showed as GnRH isoforms are also produced in the testis, and may contribute to the control of testicular germinal cell development and function [114]. Through the release and transport by circulatory system, LH and FSH can reach the target tissue: the gonads. Majorly, LH regulates the steroidogenesis in the Leydig [115] and Theca [106] cells, whereas FSH supports gametogenesis in Sertoli [89,116] and Granulosa cells [106]. In different mammalian species, upon LH stimulation, there is a feedback negative regulation in the HPG axis through testosterone release from testis, inhibiting pituitary LH production [117,118]. In contrast, LH-stimulated testosterone release from ovaries inhibits KISS production [119]. Similarly, LH-stimulated progesterone release from ovaries inhibits LH surge and pulsatile secretion that occurs at the pre-ovulatory stage through KISS upstream signaling, via progesterone receptor in the arcuate (ARC) and/or anteroventral periventricular (AVPV) nuclei in Sprague–Dawley rats [120]. FSH induces estradiol release from both testis and ovaries. While estradiol from mammalian testis represents another negative feedback mechanism on the HPG axis, at the level of GnRH and LH production [118], estradiol from ovaries can both inhibit (at the ARC) or stimulates KISS production (at the AVPV), as demonstrated in murine models [121]. Upon FSH stimulation, secreted inhibins from both mammalian testis and ovaries may also represent another major negative feedback mechanism on the HPG axis acting at the pituitary FSH production [106,117]. When considering the reported in vivo VK induction of testosterone synthesis and release from rodents and fish gonads to blood stream (above-mentioned) [49,54,55,100], it is a key point on the HPG axis to count on.

Hypothalamic neurons synthesizing KISS play a key role in the central regulation of the timing of puberty onset and reproduction in mice [122]. Saedi and co-authors [112] reviewed the tight control of the KISS signaling through neurotransmitters and neuropeptides at the human and rodent hypothalamus. Leptin, synthesized in adipocytes, is a permissive factor for puberty onset and that signaling is increased by androgens in female rats [123]. Leptin acts on KISS-positive neurons indirectly, through the cocaine- and amphetamine-regulated transcript (CART), a potent stimulator of neurons expressing KISS and GnRH, as demonstrated by the high fat diet-induction of early puberty in mice [124]. At the same time, leptin inhibits the activity of agouti-related peptide (AgRP)-expressing neurons [125], which are other cell regulators of female fertility in mouse [126]. AgRP-expressing neurons also express γ-aminobutyric acid (GABA) and neuropeptide Y (NPY), and they are tightly connected to both ARC and AVPV nuclei. Indeed, the ablation of these neurons in mice lacking leptin restored mice fertility [126]. NPY is also known to stimulate KISS signaling [127] as well as other neuropeptides, such as tachykinin-3 (TAC3, cleaved into neurokinin-B (NKB) chain) [128], as demonstrated by in vitro and in vivo assays with mouse model. Additional signals may also be involved, including the glutamate. Glutamate is the principal excitatory neurotransmitter of the central nervous system (CNS) and a known co-factor to facilitate KISS signaling [129]. In contrast, different studies in mammals have proved that high prolactin (PRL) levels and dynorphin inhibit the KISS signaling pathway [130,131]. Once secreted, KISS binds its G-protein coupled receptor on GnRH-positive neurons to stimulate GnRH synthesis in mammalian species (in [121]). It is not yet clear the role of GABA and glutamate transmissions on the KISS/GnRH signaling regulation, and it might differ in various physiological stages, as demonstrated in mice and ewe models [132,133]. GnRH neurons are surrounded by many populations of GABAergic and glutamatergic neurons, which neural transmission impact on the KISS/GnRH signaling. Indeed, estradiol induced changes in GABAergic and glutamatergic transmission may be involved in multiple aspects of negative and positive feedback on the GnRH neuronal activity in mice [132,134]. Nevertheless, exposure to agonist and antagonists of GABA receptors suggested that GABA is an important mechanism that may be involved in the biosynthesis of GnRH and GnRH receptor as well as in the GnRH release in mice and female ewes [133,135].

The impact of VK on neuronal development and homeostasis should not be also neglected when considering the tight control of brain over reproductive system through the HPG axis. GAS6 also has a central role in the development and survival of nervous system. As previously commented, GAS6 activates the TAM family of receptor tyrosine kinases, particularly involved in the proliferation and survival of GnRH neurons, allowing their migration from the olfactory bulb to the hypothalamus, as demonstrated by in vitro assays (reviewed in [51]). Furthermore, different transcripts of *pxr* were also specifically found to be expressed in the brain and pituitary in both genders from different vertebrate species [18,20,37,78,93]. Through in situ hybridization (ISH), we found a particular *pxr* transcript (*sspxr1*) in Senegalese sole to be expressed in the granular layer [20], a densely packed layer containing mossy fibers, the cell bodies of granule cells, uni-polar brush cells, and Golgi cells. This layer releases the neurotransmitter glutamate [136] and it represents a complex communication network between its own cells and those from other brain (outer) layers (Purkinje and molecular layers). Therefore, this might represent another hypothetical pathway by which VK might influence the cellular responses that are involved in the HPG axis control of the reproductive system.

On another hand, GnRH signaling is repressed by gonadotropin inhibitory hormone (GnIH) in rat and sheep females (in [121]). Recently, GnIH have been also found to regulate testicular development and function through changes in steroidogenesis and controlling GnRH-induced response in vitro [137]. GnRH signaling in female mice is also repressed by galanin (GAL), which inhibits the activity of GnRH (in [112]), while serotonin inhibits *gnrh* expression in female mice [138]. Because any neurobiological alteration on these players changes the GnRH neuronal activity needed to generate the GnRH surge and ultimately trigger ovulation, different environmental or intrinsic clues might indirectly affect HPG axis and reproductive performance. Pulsatile secretion of GnRH towards the gonadotropic cells at the anterior pituitary stimulates LH and FSH synthesis. GnRH exerts its action through its receptor (GnRHR), a seven-transmembrane receptor that is associated with two different G proteins, G_q/11_ and G_s_ (demonstrated in primary pituitary cell culture, in vivo murine models, and immortalized cell culture, reviewed by [113]). High GnRH pulse frequency preferentially activate the G_q/11_ protein stimulating phosphatidylinositol-specific phospholipase C beta (PLCβ), which in turn induced inositol trisphosphate (IP_3_) and diacylglycerol (DAG) synthesis. The former stimulates Ca^2+^ release from the smooth ER, whereas the latter leads to protein kinase C (PKC) activation. Ca^2+^ and PKC-mediated the activation of calcium/calmodulin-dependent kinase II (CAMK II) and (mitogen-activated protein kinase) ERK 1/2, respectively, finally inducing *lh* gene expression. Conversely, when GnRH is pulsed with low frequency, cyclic adenosine monophosphate (cAMP) production occurs through G_s_ protein recruitment. cAMP increased intracellular levels, activate protein kinase A (PKA), and consequently cAMP response element binding protein (CREB) phosphorylation that enhance the *fsh* gene expression.

These intracellular communications and signaling pathways might be also altered by VK. VK also induced sphingolipid synthesis, a major class of lipids and a vital modulator of neural cell proliferation, differentiation and survival in mammals (reviewed in [51]). We have recently reported how VK dietary supplementation in male Senegalese sole induced a differential expression of some particular ncRNAs in blood plasma. A bioinformatic prediction of the targets of these ncRNAs identified several genes that were specifically involved in sphingolipid (*lpin3*) and glycerophospholipid (*cept1b* and *lpin3*) metabolism and/or signaling, in addition to *calcium/calmodulin dependent protein kinase II beta* (*camk2b1*) gene [49]. While lipins (Lpin1, 2, and 3) are known to regulate fatty acid metabolism, particularly the conversion of phosphatidic acid to DAG; Cept1 catalyzes the *de novo* synthesis of phosphatidylcholine (PC) and phosphatidylethanolamine (PE) from DAG, critical for homeostasis of cellular lipid stores and membranes, but also to induce the expression of LH in the pituitary, as demonstrated by in vitro studies [113,139,140]. Indeed, the release of Ca^2+^ from the ER controls the activation of calcium/calmodulin kinase I (CAMKI) and II (CAMKII), as well as cell junctions at pituitary, Leydig and Sertoli cells might be an additional pathway where VK may act through these ncRNAs targeting *camk2b1*.

In mammalian testis, LH binding to luteinizing hormone/choriogonadotropin receptor (LHCGR) induces cAMP and cyclic guanosine monophosphate (cGMP) increase in Leydig cells. Consequently, PKA, PKC, and protein kinase G (PKG) activity is stimulated, as well as Ca^2+^ released from ER. While Ca^2+^ release activates CAMKI, PKA and PKC induce the expression of several steroidogenic genes through ERK 1/2 signaling pathway, in particular: *steroidogenic acute regulatory protein* (*STAR*), *cholesterol side-chain cleavage enzyme (*also known as *cytochrome P450 11a1; CYP11A1*), *steroid 17-alpha-hydroxylase/17,20 lyase* (also known as *cytochrome P450 17a1; CYP17A1*), *3 beta-hydroxysteroid dehydrogenase/Delta 5-->4-isomerase type 1* (*HSD3B1*), and/or *testosterone 17-beta-dehydrogenase 3* (also known as *17-beta-hydroxysteroid dehydrogenase type 3*; *HSD17B3*) [115]. In Sertoli cells from mammals, FSH induces Ca^2+^ release at ER and stimulates PKA and phosphatidylinositol 3-kinase (PI3-K) through cAMP. While Ca^2+^ release from ER regulates Sertoli–Sertoli cells junction communication, PKA activation stimulates the expression of *aromatase* (also known as *cytochrome P450 19A1*; *CYP19A1*), *tissue-type plasminogen activator* (*PLAT*), *insulin like growth factor 1* (*IGF-1*), and *stem cell factor* (*SCF*) genes that potentiates spermatogonia survival. Furthermore, PI3-K stimulates *γ-glutamyl transpeptidase* (*γ-gtp*) and *lactate dehydrogenase* (*ldh*) expression, the latter being involved in the lactate production for germ cells development. In addition, FSH also activates phospholipase A2 (PLA2) pathway, leading to prostaglandin E2 (PE2) release [116]. In the mammalian ovary, at the antral follicles, LH preferentially target Theca cells to stimulate steroidogenesis, increasing the gene expression of *star*, *cyp11a1*, *hsd3b1, hsd3b2*, and *cyp17a1* through PKA signaling and producing androgens and progesterone (P4). Instead, in the Granulosa cells of antral follicles, FSH binds to its receptor, activates PKA and PI3-K signaling, inducing activins, inhibins, and follistatin, as well as supporting androgen aromatization to estrogens, oocyte growth, and granulosa cells proliferation through increasing the expression of *cyclins*, *cyclin-dependent kinases*, *aromatase*, and *cyp17a1*. In parallel, the expression of *lhcgr* is also induced [141]. At the pre-ovulatory stage, LH surge acts in the Granulosa cells to regulate the final steps of folliculogenesis [106,142]. In this sense, PKA and PKC signaling pathways are activated in order to induce maturation-promoting factor (MPF), chondroitin sulfate proteoglycan 2 (CSPG2), proteolytic system, and epidermal growth factor-like factors (EGF-like factors), as well as the expression of *progesterone receptors* (*pr-a* and *pr-b*).

### 3.3. The Vitamin K Impact on Tissue Crosstalk Relevant for Reproductive Performance

**Figure 5:**
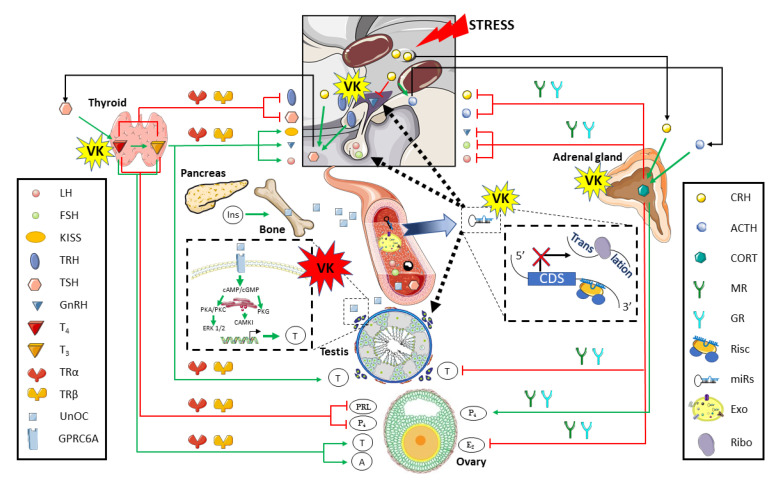
Tissue crosstalk in vertebrate gametogenesis and gamete quality. On the left hand, the crosstalk between the thyroid gland and the pancreas-bone with the hypothalamus-pituitary-thyroid (HPG) axis is depicted. On the right hand, the respective crosstalk between the stress response system and the adrenal gland with the HPG axis is presented, while the crosstalk of HGP and other tissues through the circulating non-coding RNAs (ncRNAs) whether included in exosomes or not is hypothesized. The particular mechanisms by which vitamin K (VK) has a demonstrated effect on reproductive tissue crosstalk are highlighted in red, while those showing potential evidences are highlighted in yellow. A, androgens; ACTH, adrenocorticotropic hormone; CDS, coding sequence; CORT, glucocorticoids; CRH, corticotropin releasing hormone; Exo, exosomes; E_2_, estradiol; D1, deiodinase type 1; D2, deiodinase type 2; D3, deiodinase type 3; FSH, follicle-stimulating hormone; GnRH, gonadotropin-releasing hormone; GPRC6A, G Protein-Coupled Receptor Class C Group 6 Member; GR, glucocorticoid receptor; Ins, insulin; KISS, kisspeptin; LH, luteinizing hormone; miRs, microRNAs; MR, mineraloglucocorticoid receptor; PRL, prolactin; P_4_, progesterone; Ribo, ribosomal subunits; Risc, RNA-induced silencing complex; T, testosterone; TRα, thyroid hormone receptor alpha; TRβ, thyroid hormone receptor beta; TRH, thyrotropin-releasing hormone; TSH, thyroid-stimulating hormone; T_4_, 3,5,3′,5′-tetraiodothyronine; T_3_, 3,5,3′-triiodothyronine; UnOc, uncarboxylated osteocalcin.

The crosstalk of some peripheral organs/tissues with the reproductive system is important to regulate steroidogenesis and gametogenesis. Figure 5 depicts the main tissue crosstalk points that are relevant for reproduction success. In vertebrates, the thyroid gland exerts a key crosstalk regulation of reproduction through the synthesis and release of thyroid hormones (THs), triiodothyronine (T_3_), and thyroxine (T4; reviewed in [143]). THs concentration is critical for brain development during the embryonic development and neonatal life, but also in energy metabolism, lipid synthesis and degradation, linear growth, bone maturation, and remodeling (as reviewed in [143]). Besides, there is a clear crosstalk between HPG axis and THs. Thyrotropin-releasing hormone (TRH) is synthetized in the hypothalamus, which, in turn, regulates the thyroid-stimulating hormone (TSH) release from the anterior pituitary. The TSH target organ is the thyroid gland and/or follicles, where induces T_4_ synthesis and that can undergo deiodination to T_3_ through deiodinases (D1, D2, and D3; reviewed in [144]. How THs interact with the reproductive system may differ between vertebrate species, and particularly in fish species (reviewed in [90]). While TRH is involved in the activation of pituitary-thyroid axis in mammals, this seems to not occur in fish. Further, the pituitary secretion of TSH in fish seems to be dependent on corticotropin-releasing hormone (CRH), as observed in amphibians, reptiles, and birds.

In mammals, once released to the blood stream, THs can be transported associated with globulin, transthyretin, and albumin [145] and thus, reaching target tissues and exerting their own known actions by binding to their NRs (thyroid hormone receptors; TRs). Elevated THs blood levels exert a negative feedback, inhibiting TRH and TSH release in a diverse set of vertebrates, including human, rat, bird and sheep, among other animals [146]. Silva and coauthors, in 2018 [147], reviewed thyroid hormones involvement in female reproduction in humans and other vertebrates, highlighting the thyroid hyperfunction association with androgens and testosterone synthesis, which then are metabolized to estrone and E_2_, respectively, suggesting a positive THs control of aromatizable steroids in the ovary. In contrast, some studies evidenced a negative relationship between hypothyroidism and increased serum concentration of P_4_ and PRL, which cause a luteal phase prolongation, as demonstrated by hyperthyroidism induced premature luteolysis in rats (reviewed in [147]). In male tilapia (*Oreochromis niloticus*), T_3_ stimulates *kiss* and *gnrh* gene expression (reviewed in [147]), while hypothyroid conditions mediate a decrease of LH and testosterone levels in cockerel and rat (reviewed in [148]). In teleost fish, THs are associated with testicular development, growth, and maturation, through the promotion of germ cell proliferation and differentiation [90]. THs impact sexual differentiation and reproductive performance relies on the gene expression regulation of key steroidogenesis genes, such as *androgen receptor* (ar), *steroid 5α-reductase 1* (*srd5alpha1*) and *2* (*srd5alpha2*) *aromatase* (*cyp19a1*), as *anti-Müllerian hormone* (*amh*) and consequently estradiol levels in all vertebrates, as well as *insulin-like growth factor binding protein 1* and *3* (*igfbp1a* and *igfbp3*), particularly in zebrafish [90].

Stress conditions are also important for reproductive success. Under stress, vertebrates increase the production of CRH at the hypothalamus that will stimulate the synthesis of the adrenocorticotropic hormone (ACTH) in the anterior pituitary. In humans, ACTH induces steroidogenesis [149], but also induce glucorticoids (CORT) synthesis in the adrenal cortex. This is the basis of the hypothalamus-pituitary-adrenal gland crosstalk, although CRH can also directly stimulate the release of CORT from adrenal gland (reviewed in [150]) as well as inhibit GnRH secretion in the rhesus monkey [151]. Moreover, the fact that CRH can also stimulate the TSH synthesis clearly shows how complex might be the crosstalk regulation of the reproduction through peripheral tissues (reviewed in [143]).

CORT acts on target tissues through mineralocorticoid receptor (MR) and glucocorticoid receptor (GR). Besides increasing blood glucose levels, acting on cardiovascular and immune system with anti-inflammatory properties, CORT is also involved in human and murine reproduction (reviewed in [152]). Increased adrenal CORT levels exert negative feedback through the inhibition of the entire HPG axis. At central level, it is known that CORT had an inhibitory effect on GnRH and LH release, whereas its potential effect on FSH results are somehow contradictory (reviewed in [153]). In male rats, stress-induced CORT release also has a direct inhibitory effect on testosterone synthesis in Leydig cells [153,154], in addition to the impact at hypothalamic and pituitary level. At the mammalian ovary, CORT is known to exert both agonist and antagonist effects, since an increase of the basal CORT level during the ovulatory process is needed, whereas stress-induced CORT release inhibits E_2_ but increases P_4_ levels [153,155].

Although significant levels of several K vitamers (particularly MK-4) were found in tissues, like mammalian thyroid and adrenal gland [9,16], and *ggcx*, *vkorc1* and *vkorc1l1* were reported to be expressed in the adrenal gland of male and female mice [92], little is known regarding how VK might affect thyroid and adrenal hormones signaling. While PXR-regulated transcriptome is dependent on the nutritional status, affecting thyroid hormone metabolism II in mice [156], several studies have shown that PXR plays an important role in adrenal steroid hormone homeostasis. Activation of PXR markedly increases plasma concentrations of corticosterone and aldosterone, the primary glucocorticoid and mineralocorticoid in rodents, being associated to the activation of adrenal steroidogenic enzymes, including CYP11A1, CYP11B1, CYP11B2, and HSD3B2 [157]. In a vertebrate’s model for developmental biology, the zebrafish, the exposure to warfarin during embryogenesis affect the thyrotropin-releasing hormone receptor signaling pathway, at least at the transcriptomic level [41], suggesting that the VK status might influence THs synthesis and homeostasis.

The pancreas-bone-testis axis communication is the most characterized tissue crosstalk by which VK might exert an important role in vertebrate’s reproduction. At the pancreas, within the islets of Langerhans, β cells produce insulin, an anabolic hormone, promoting carbon energy deposition in the body and participating in the control of glycemia [158]. A failing control of glycemia through insulin induce complications, such as neuropathy, nephropathy, retinopathy, vascular and bone disorders in vertebrates. Regarding the effects on skeletal tissue, humans with type 2 diabetes suffer higher fracture risk [159]. Indeed, insulin stimulates osteoblast differentiation favoring osteoclast bone resorption and generating and releasing undercarboxylated OCN in mouse models [50]. OCN is a VKDP shown to control bone mineralization, as demonstrated in OCN-deficient mice [160]. Once carboxylated, it has a high affinity for calcium and hydroxyapatite, the mineral component of the bone extracellular matrix [161], but, during bone remodeling, the acid pH produced by osteoclast favors its undercaboxylation status and release to circulation, enhancing male fertility (reviewed in [162]). *ocn*^−/−^ mice are poor breeders that are characterized by low testosterone levels, oligospermia and a reduced reproductive organ weight, a condition that can be reversed by OCN administration [163]. OCN may act on Leydig cells through the G protein-coupled receptor class c group 6 member (GPRC6A). *Gprc6a*^−/−^ and *ocn*^−/−^ mice have almost identical metabolic and reproductive phenotypes, including reduced testosterone levels and particularly, Leydig cell-specific deletion of GPRC6A (*gprc6a*Leydig+/−) leads to these reproductive defects in males [164]. OCN signaling regulates the expression of genes that are associated with testosterone production in a cAMP response element binding protein (CREB)-dependent manner, as demonstrated by CREB phosphorylation in osteocalcin-treated Leydig cells and corroborated in *Creb_Leyding_^−/−^* mice [163]. Indeed, unOCN has been shown to activate GPRC6A using Docking simulation and in vitro assay with MA-10 and β-TC6 cell lines [165], inducing the PKA, PKC, PKG activation, as well as the Ca^2+^ release [115], driving the specific expression of *star*, *cyp11a*, *cyp17*, and *3β-hsd* genes that are required for testosterone biosynthesis, and representing a complex pancreas-bone-testis axis communication (reviewed in [162,166]). Interestingly, it was recently found that this regulation through OCN was LH independent in Sprague-Dawley male rats [167]. Moreover, because PXR-deficient rodents had disrupted glucose homeostasis [168], a low transactivation of PXR due to VK deficiency might be an additional source of altered glucose homeostasis and thus, male reproductive disorders.

With the discovery of the key role of non-coding RNAs (ncRNAs) in cell biology, a diverse set of ncRNAs (e.g., micro-RNAs (miRs) and P-element–induced wimpy testis (PIWI)–interacting RNAs (piRs)) involved on vertebrate’s reproduction as well as their corresponding DNA/mRNA target sequences were identified. A key role of piRs in male gametogenesis was clearly evidenced by the male sterility in mice lacking the piR pathway [169]. Similarly, different miRs have been shown to have great influence on cell mitosis during mammalian gametogenesis (reviewed in [170]). In fish species, like salmon, not only new specific miRs have been linked to testis maturation, but also a functional conservation in vertebrates of known miRs involved in the transition into puberty have been suggested [171]. Different clinical studies have been explored the use of miRs as potential biomarkers for human male factor infertility [104,172]. Furthermore, in humans, the presence of specific miRs (such as miR-31) in seminal plasma exosomes might be not only a suitable biomarker of azoospermia, but also a prognostic tool of embryo development [172]. In a recent study, dietary VK supplementation during six months in Senegalese sole not only increased testosterone plasma levels in both genders and improved male’s sperm quality (above-mentioned), but also specific ncRNAs differentially expressed in blood plasma through RNA-Seq analysis were discovered [49]. In these males, a lower expression of *miR-146* family members (*miR146a-1-2-3* and *miR-146a*) and a higher of *let-7* family members (*let-7g*, *-7e* (18 nt), *-7a1, -7a-3, -7a-2, -7a-1, -7e* (23 nt)) and *piR-675//676//4794//5462* was found when compared to that of males fed the Control diet (with higher sperm DNA fragmentation). Both *miR146* and *let-7* family members are expressed in spermatogonia and only *let-7s* in spermatocytes, and oppositely involved in the RA-induced spermatogonia differentiation in mice [173,174]. A bioinformatic analysis predicted some genes involved in glycoprotein hormone α-subunit synthesis and GnRH pathway (among other physiological processes), as mRNA targets of those ncRNAs, suggesting a further more complex regulation of reproduction in Senegalese sole males, and clearly associated with VK [49].

## 4. Conclusions

Fulfilling the dietary recommended intake (DRI) of each nutrient is basic for a proper development and health, and to complete all of the required biological functions, including reproduction. Furthermore, not covering the requirements for a particular nutrient may have deeper impact on the progeny, as it has been found for VK in different vertebrate species [49,175]. Knowledge on VK metabolites, metabolism, functions, and identification of suitable and accurate biomarkers is critical to identify the DRI for VK in vertebrates. Nowadays, VK DRI is based solely on the intake of vitamin K1 and the quantification of blood coagulation time and/or the uncarboxylated forms of BGP and MGP [24]. Based on the literature here reviewed, it seems more appropriated to define DRI considering biological process other than blood coagulation, including reproduction. Furthermore, taking into account the potential role of VK2 in neural and gonad development, a specific DRI on VK2 might be highly advisable as well as the use of different and specific biomarkers for fixing the nutritional requirements of VK in vertebrate’s gametogenesis.

In particular, VK seems to have an impact on testosterone synthesis among other steroids, oxidative stress response, and osteocalcin signaling in gonads, as well as GnRH, glutamate, and intracellular calcium levels and signaling at hypothalamus and pituitary tissues. While some classical biomarkers of gametogenesis, such as testosterone, LH, and FSH serum levels, are already available for determining the optimal VK dietary level for vertebrate’s reproduction, a better understanding of the precise signaling pathways active in the organs, and tissues that are involved in the complex regulation of reproduction would allow a deeper and complete prediction of the effects elicited by VK nutritional status. Recent results on the potential cell-to-cell communication through circulating ncRNAs revealed an unexpected regulatory pathway, by which VK may exert a role on reproductive performance. Future research on this issue, as well as on the potential role of VK on thyroid and adrenal gland, might bring new light to understand the broad biological role of VK in vertebrates. Last, but not the least, we have recently shown as the use of fish teleosts is an interesting biological model in vertebrates to identify the effects and describe the conserved VK-related molecular pathways by which VK has a great impact, not only in blood coagulation and bone tissue, but also in reproduction. In this sense, the use of these species might help to unveil whether parental VK nutritional status might determine primordial germ cell population (PGCs), sex gender, gonad development, and/or reproductive performance on their progenies.
